# The influence of Pseudomonas aeruginosa infection on the airway metabolome

**DOI:** 10.1099/mic.0.001617

**Published:** 2025-10-08

**Authors:** Angharad E. Green, Dilem Ruhluel, Marie Phelan, Joanne L. Fothergill, Daniel R. Neill

**Affiliations:** 1 Advanced Research Computing Centre (ARC), University College London, London, UK; 2 Department of Clinical Infection, Microbiology and Immunology, University of Liverpool, Liverpool, UK; 3 Highfield NMR Facility, Liverpool Shared Research Facilities (LIV-SRF), University of Liverpool, Liverpool, UK.; 4 Department of Biochemistry and Systems Biology, Institute of Molecular, Systems and Integrative Biology, University of Liverpool, Liverpool, UK; 5 Division of Molecular Microbiology, School of Life Sciences, University of Dundee, Dundee, UK

**Keywords:** metabolomics, NMR, *Pseudomonas aeruginosa*, respiratory infection

## Abstract

*Pseudomonas aeruginosa* is an environmentally resilient bacterium and an important cause of both acute and chronic infections in people with impaired natural barriers or immunological defences. Chronic respiratory infection with *P. aeruginosa* is a major cause of morbidity and mortality in people with airway diseases, including cystic fibrosis (CF) and non-CF bronchiectasis. Chronic airway infection is characterized by periods of relative stability punctuated by pulmonary exacerbations, during which times rapid bacterial outgrowth necessitates intense antimicrobial chemotherapy. The periods of stable infection can be modelled in mice by nasal instillation of airway-adapted *P. aeruginosa* in saline, leading to prolonged colonization of both upper airway (sinus) and lower airway (lung) environments that is not associated with symptomatic disease. Here, we use NMR metabolomics to investigate the impact of *P. aeruginosa* colonization on the metabolic landscape of sinuses and lungs. Lung infection led to pronounced changes in the airway metabolome, with significant depletion of glucose and myo-inositol but enrichment of glutathione (GSH), relative to uninfected lungs. Changes in the sinuses were more subtle but could be identified through dimensionality reduction approaches. The NMR spectral peaks that discriminated between infected and uninfected sinuses in partial least squares discriminant analysis included those for lactate and choline but were mostly representative of yet unidentified metabolites. These data highlight the differential impact of infection on separate airway compartments and identify undefined metabolites undergoing pronounced abundance changes during infection.

## Introduction

The airways are repeatedly exposed to inhaled environmental micro-organisms. In health, this results in minimal perturbation to the immunological and metabolic homeostasis of the respiratory environment, which is maintained by a delicate balance of host and commensal microbial processes [1]. However, disruption of this balance, by colonization of the airways by an opportunistic pathogen, or through the inflammatory processes associated with chronic respiratory disease, can result in rapidly deteriorating lung health.

*Pseudomonas aeruginosa* is not typically considered an airway commensal but is commonly isolated from airway samples of those with chronic respiratory diseases, including cystic fibrosis (CF) and non-CF bronchiectasis [2]. Studies in people with CF have demonstrated that chronic airway infection with *P. aeruginosa* is not restricted to the lungs, with the paranasal sinuses acting as an important microbial reservoir [3,6]. Isolates from sinuses and lungs are often genetically indistinguishable [7,8], and it has been proposed that upper airway environments – including the sinuses – act as protective niches within which *P. aeruginosa* might adapt to the respiratory environment before seeding down into lungs [9,10].

In a previous study, we used NMR analysis of samples from mouse infection models to profile the metabolic landscape of the airways in conditions of health and of respiratory infection [11]. Using *Streptococcus pneumoniae*, a human airway commensal and opportunistic pathogen, we identified that infection can profoundly impact the metabolic profile of respiratory tract tissues. Furthermore, we showed that upper and lower airway environments have distinct metabolic features and that infection differentially affects the two niches. Here, we sought to determine whether the infection-induced changes we observed were generalizable host responses to microbial colonization or whether the microbial species and the clinical trajectory of the infection were important determinants of the ensuing metabolite shifts.

The experiments described herein differ from those performed with *S. pneumoniae* in two important respects. Firstly, we here use *P. aeruginosa*, which, unlike *S. pneumoniae*, is not an airway specialist. Whilst the evolution of *S. pneumoniae* has been towards an ever more intimate association with host airway environments, *P. aeruginosa* is a predominantly environmental organism and a generalist species, with a large and plastic genome. Secondly, in the mouse models used for these experiments, lung colonization with *S. pneumoniae* leads to induction of strong inflammatory responses that result in either clearance of infection or symptomatic pneumonia, whilst lung colonization with *P. aeruginosa* leads to prolonged, sub-clinical infection that is not associated with overt signs of inflammation. Our findings reveal both commonalities and differences in metabolic profile alterations invoked by infection with the two pathogens.

## Methods

### Strains and culture conditions

Liverpool Epidemic Strain B65 (LESB65) was used throughout. The LESB65 strain was chosen for this study as it colonizes the mouse airways readily, giving rise to a characteristic infection progression [6]. For preparation of infection stocks, bead stocks from the −80 °C freezer were streaked onto lysogeny broth (LB) agar plates (Oxoid, UK), grown overnight at 37 °C and then a loop of colonies was transferred into 5 ml LB. This was cultured overnight at 37 °C and then a subculture was established in fresh LB the next day. Cultures were grown to mid-exponential phase (OD_600_ 0.4–0.6) and then divided into 500 µl aliquots for storage at −80 °C. After at least 48 h in the freezer, two aliquots were removed, pelleted, washed in PBS and then bacterial numbers quantified by serial dilution onto LB agar. The calculated bacterial density per stock tube was used to determine dilutions for subsequent infection experiments.

### Ethics statement

All animal infections were performed at the University of Liverpool, with prior approval from the UK Home Office (project licence PP2072053) and the local animal welfare ethical review board. The principles of the Declaration of Helsinki were observed throughout. Mice were housed in individually ventilated cages, with access to food and water *ad libitum*. Environmental enrichment was provided in all cages, and mice were acclimatized to the animal unit for at least 7 days before use. Mice were randomly allocated to cages on arrival in the animal unit by staff with no role in study design. For experiments reported in this manuscript, individual mice were considered the experimental unit. Sample sizes, controls and statistical analyses are detailed in the figures and accompanying legends. Any samples excluded from analyses following quality control testing are detailed in the manuscript text.

### Mouse infections

Female BALB/c mice (Charles River, UK) of 6–8 weeks of age were used. Mice were infected with 2×10^6^ colony forming units (c.f.u.) of LESB65 in 50 µl PBS. Infection was achieved by dropwise addition of the inoculum to the nares of lightly anaesthetized mice, allowing for natural inhalation. A mix of oxygen and isoflurane was used for anaesthesia. Following infection, mice were monitored for signs of disease and culled at pre-determined time points for tissue collection. Tissues were used for infection burden determination by serial dilution of homogenates onto Pseudomonas selective agar, or for metabolite extraction (48 h timepoint only).

### Tissue dissection

To avoid batch processing effects, tissues were harvested from two mice per experimental group at a time until all mice were culled and upper airway tissue and lungs harvested. Upper airway tissue was sampled according to the revised guides for organ sampling and trimming in rats and mice [12]. Recovered upper airway samples included both sinus and nasopharyngeal tissue, referred to throughout the manuscript collectively as ‘sinuses’. The intact sinus and lung samples were dipped in ice-cold PBS solution, pH 7.4, to remove any blood surrounding the organ. Tissues were placed into 1.5 ml bead beating tubes, snap frozen in liquid nitrogen and stored at −80 °C until metabolite extraction (for a period of no more than 3 months).

### Metabolite extraction

Metabolite extractions were carried out in a random order to minimize batch effects. Each frozen sample was re-suspended in 0.8 ml ice-cold extraction solvent [50:50 (v/v) acetonitrile/water]. A bead beating protocol was used to homogenize the tissues for metabolite extraction, using 0.5 g of 2.8 mm diameter ceramic beads for lungs and 0.25 g of beads for sinuses. Each tube was bead bashed for 3 min, using a BeadBug tissue homogenizer (Sigma Aldrich, Gillingham, UK), in cycles of 1 min with tubes put on ice between cycles. This process was carried out in a cold room, maintained between 2 and 4 °C. After homogenization, the lysate was transferred to fresh 1.5 ml microfuge tubes and centrifuged at 4 °C for 10 min at 21,500 *g*. Supernatants were transferred into fresh 1.5 ml microfuge tubes, snap frozen in liquid nitrogen and stored at −80 °C until they were transferred on dry ice to the University of Liverpool NMR Centre for lyophilization and NMR profiling. Cell pellets were frozen down and stored at −80 °C.

### NMR profiling

Supernatants from extracted samples were processed at the University of Liverpool NMR centre. These were lyophilized overnight at −55 °C and processed before NMR sample preparation and NMR acquisition. NMR samples were resuspended in 200 µl 100 mM phosphate, pH 7.4 (100% 2H_2_O) and transferred to 3 mm outer diameter NMR tubes prior to acquisition on a 700 MHz Avance III HD spectrometer equipped with a TCI cryoprobe (Bruker, Massachusetts, USA). Quality assurance of the spectrometer followed best practice [13,14] with temperature stability within 0.1 °C using a deuterated methanol thermometer [15] and 3-dimensional shimming on a standard reference sample (2 mM sucrose), prior to sample acquisition, to ensure spectrometer optimization. Samples were acquired using vendor supplier pulse sequence (cpmgpr1d) and automated acquisition and processing routines for maximum consistency.

Spectral acquisition was performed using a standard ^1^H 1D Carr Purcell Meiboom Gill pulse sequence with 256 transients. All parameters are available with the deposited data in the EBI repository MetaboLights (MTBLS6052) [16]. Spectra were automatically processed with phasing, Fourier transformation and window function through vendor supplied routine (apk0.noe). Alignment was performed manually to the alanine CH3 doublet at 1.55 p.p.m.

### Data analysis

Spectra were binned interactively using an in-house workflow within Galaxy server toolkit tameNMR (https://github.com/PGB-LIV/tameNMR). Metabolites were matched to an in-house library (supplemented with annotation from the Chenomx v8.2 mammalian metabolite library). TopSpin (v4.1.3) was used to measure the peak boundaries to develop the pattern file from an existing pattern file, C2C12, as described in our previous analysis of murine respiratory tissue [11]. All statistical analysis was carried out using R statistical software (v4.1.0) with in-house R scripts provided by the University of Liverpool computational biology facility (https://www.liverpool.ac.uk/computational-biology-facility) to perform established univariate and multivariate analyses [17,18]. Briefly, binned NMR spectrum datasets were normalized by probabilistic quotient normalization (PQN) [19] before univariate analysis via one-way ANOVA with Bonferroni (Bonf) multiple testing correction and 0.05 significance level and Tukey’s simultaneous test for difference of means post-hoc analysis. Fold changes were calculated with respect to the sham (PBS) infected control and presented as natural logs to indicate an increase (positive value) or decrease (negative value) of each metabolite in a given group. Prior to in-depth analysis of data, we compared PQN to other methods of normalization (tissue mass normalization and total intensity normalization) and found that the three methods produced comparable outputs. Metabolite peaks were scaled using the Pareto method prior to multivariate analysis. Unsupervised principal component analysis (PCA) was performed to observe the main sources of variance between samples, and supervised partial least squares discriminant analysis (PLS-DA) was used to determine predictive models for metabolite profiles. PLS-DA plots were cross-validated against 30% randomly excluded samples to establish model quality as area under the receiver operating curve (AUROC) [20]. Ranked variable importance in projection (VIP) scores >1 inferred which metabolites were influential within each PLS-DA model.

## Results

### Airway colonization by *P. aeruginosa* triggers changes to the metabolic landscape

**Fig. 1.**
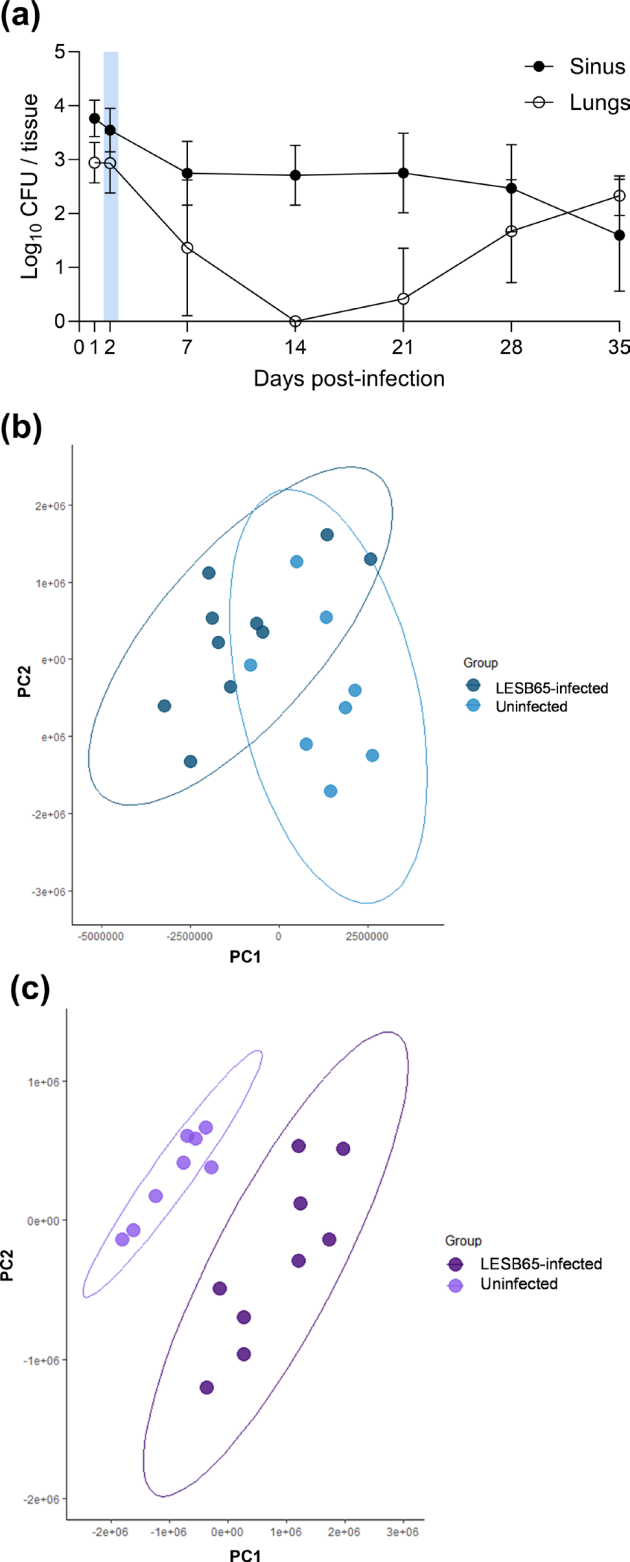
Metabolic changes in the upper and lower airways in a mouse chronic *P. aeruginosa* infection model. (a) Trajectory of infection (c.f.u.) in sinuses and lungs of mice infected with *P. aeruginosa* LESB65. Blue shading shows the 48 h post-infection time point chosen for NMR metabolomics analysis. Error bars are sd of *n*=5 mice per timepoint. PCA of NMR spectra derived from (b) sinus tissue or (c) lung tissue of mice infected with LESB65- or sham-infected with PBS at 48 h post-infection. Ellipses indicate a 95% confidence level. *N*=10 for LESB65-infected sinuses, *n*=9 for LESB65-infected lungs and *n*=8 for sham-infected tissues.

We recovered paired upper airway (nasopharynx and sinus) and lung samples from mice infected with a Liverpool Epidemic Strain (LES) isolate of *P. aeruginosa*. In this natural inhalation model, airway colonization lasts upwards of 1 month but is not typically associated with symptomatic disease. The characteristic progression of infection is of early colonization of both sinuses and lungs, followed by clearance of lower airway infection (or suppression of infection below detectable limits), maintenance of a silent bacterial reservoir within the sinuses and eventual re-emergence of bacteria in lungs [6] (Figs 1a and S1, available in the online Supplementary Material). We chose an early timepoint of 2 days post-infection for NMR analysis, when we could be confident of recovering both upper and lower tissues with active bacterial colonization. Excised tissues underwent metabolite extraction and NMR spectroscopy, with samples taken from sham (PBS) infected mice used as comparators for subsequent analyses. An example NMR trace derived from the lungs of an LESB65-infected mouse is shown, with metabolite peaks labelled, in Fig. S2. Analysis of NMR spectra showed that whilst there was little detectable change in the sinus metabolome during infection, the metabolic landscape of lungs was significantly perturbed by *P. aeruginosa*, as highlighted by PCA of the two tissues (Figs 1b, c and S3). For comparison of NMR spectra from infected and uninfected lung tissue, 75.89% of data variance was explained by the first two principal components, rising to 88.2% with the inclusion of a third component (Table S1).

### Infection is associated with glucose and myo-inositol depletion in the airways

Ten lung NMR spectra peaks showed significantly increased abundance in LESB65-infected tissue, as compared to sham-infected tissue, after correction for multiple comparisons. NMR spectra exhibit multiple peaks per metabolite, due to the differing local chemical structure and environment of each individual hydrogen atom within the metabolite molecule. Of the ten peaks in lung spectra that were significantly increased during infection, four were identified as GSH, one as maltose, one as alanine and four were of unknown identity (Table 1). Thirty-four lung peaks showed significantly decreased abundance in infection (Table 1). Thirteen of these were identified as glucose and a further four as myo-inositol. Ten peaks were of unknown identity.

**Table 1.** Significantly differentially abundant metabolite peaks in NMR spectra from infected and uninfected airway tissue Red shading denotes peaks with significantly increased abundance in infection; blue shading represents decreased abundance in infection. The significant spectral bins column denotes the peak ID corresponding to the pattern file that can be found in File S2. Due to the nature of the analytical method, NMR spectra exhibit multiple peaks per metabolite. The total number of spectral bins column denotes the number of peaks in the pattern file that are representative of each individual metabolite.

Niche	Metabolite	Significant spectral bins	Total no. spectral bins	Log2 fold-change	Bonferroni adjusted p-value
Sinus	Unknown	312	n/a	0.156248562	0.01046
		89		0.411572587	0.02075
Lungs	Beta-amino acid	268	4	0.741999196	0.00491
	Glutathione	191	28	0.363796234	0.00055
		192		0.291343592	0.02276
		366		0.518650288	0.02667
		193		0.23920157	0.00343
	Maltose	168	9	0.256011012	0.01585
	Unknown	111	n/a	2.3141212	0.00354
		267		0.550996179	0.01686
		269		0.856456686	0.00004
		78		0.396804609	0.01954
Sinus	Myo-inositol	224	12	−0.167401286	0.01121
	Glucose	233	28	−0.518860682	0.03099
		232		−0.581780317	0.04069
		230		−0.54937935	0.04608
Lungs	Glucose	204	28	−0.47216161	0.00293
		132		−0.870082027	0.00002
		133		−0.878219135	0.00001
		181		−0.609304665	0.00196
		182		−0.478783572	0.00158
		230		−0.911983892	0.00001
		232		−0.925980605	0
		233		−0.880102264	0
		234		−0.770777302	0.00001
		240		−0.891946751	0.00003
		261		−0.894363904	0.00792
		178		−0.49126024	0.02943
	N-N-Dimethylglycine/N-acetylcysteine	292	28	−0.658727239	0.00078
	Lactate	162	4	−0.528044298	0.01113
	Maltose	124	9	−0.922672379	0
		222		−0.511340735	0.02039
	Myo-inositol	225	12	−0.439979109	0.00651
		164		−0.306893046	0.00272
		224		−0.837473227	0
		229		−0.483731731	0.00047
	Pi-Methylhistidine	207	4	−0.694736064	0
	Serine	186	6	−0.410554328	0.00057
	Pantothenate	227	4	−1.601177148	0.00555
	Tryptophan	56	5	−0.874209378	0.0127
	Unknown	116	n/a	−0.612369027	0.03691
		206		−0.75813651	0.0001
		226		−0.444182123	0.02634
		245		−0.393949614	0.02726
		294		−0.821480082	0.0433
		295		−0.835984882	0.00452
		296		−0.92477055	0.00193
		5		−0.915421173	0.00467
		57		−1.063870055	0.00119
		80		−0.645300106	0.04532

**Fig. 2.**
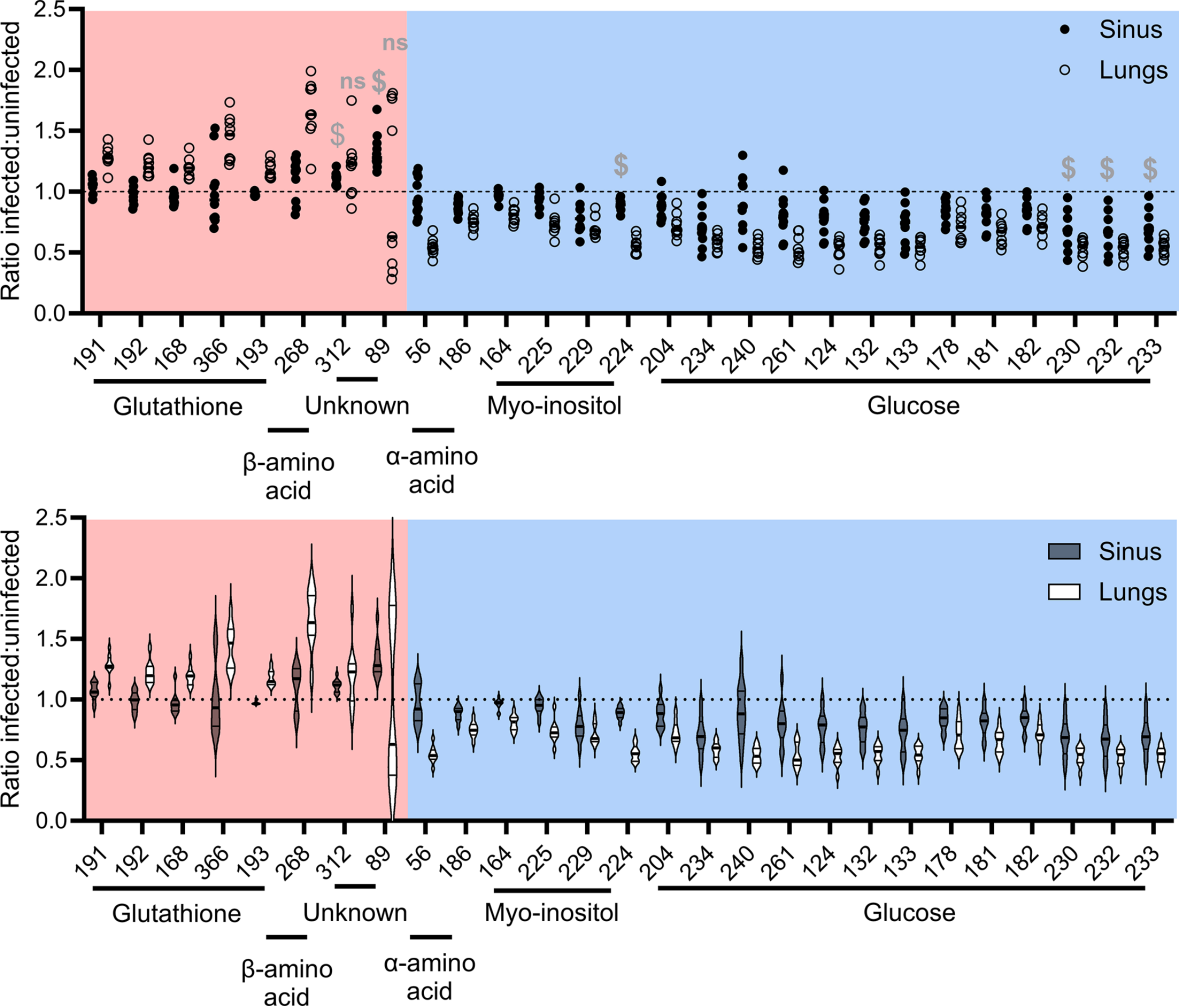
Significantly altered metabolites in *P. aeruginosa*-infected sinuses and lungs. Metabolites are listed by their peak number, of which a full list can be found in File S2. Data are presented as the ratio of metabolite abundance for infected vs uninfected tissue (i.e. infected sinus vs uninfected sinus, infected lung vs uninfected lung) and depicted as a scatter dot plot (upper panel) or violin plot (lower panel). Each datapoint is derived from a single mouse tissue. Red shading represents peaks of significantly increased abundance in infection, with blue shading representing significantly downregulated peaks. All represented peaks showed significantly differential abundance in lungs, except for the two indicated as non-significant (ns). For sinuses, only those peaks denoted with a dollar sign ($) showed significantly differential abundance. Only peaks with *P* values <0.05, after Bonferroni adjustment, are shown. Statistical analysis was determined from raw abundance data, as presented in File S2.

In sinuses, substantially fewer significant changes were observed. Only two peaks, both with unknown metabolite identities, were significantly increased in abundance during infection, whilst four peaks were associated with metabolites that decreased in abundance during infection (Table 1). All four of these peaks were also decreased in abundance in infected lungs. One represented myo-inositol and three represented glucose. Beyond these shared changes in the four peaks of decreased abundance, the changes in sinuses and lungs caused by infection were largely distinct, with many of the differentially abundant peaks from lungs not showing comparable changes in the sinuses during infection (Fig. 2).

### Signatures of metabolic change in sinuses and lungs during infection

**Fig. 3.**
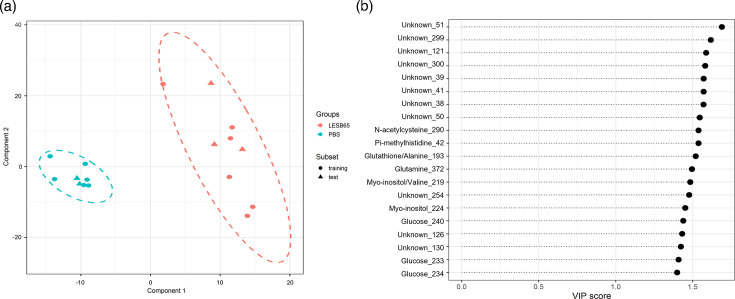
PLS-DA of lung metabolome during *P. aeruginosa* infection. (a) Multivariate PLS-DA was carried out to determine predictive models that distinguish metabolite profiles of lung tissue infected with LESB65 (red) vs sham-infected with PBS (blue). In this scores plot, the data are represented by two PLS-DA components, with the model fit to a random subset of data and evaluated on the remaining data (circles for training and triangles for test). The test examples are inside the clusters of training examples, indicating a good model fit. (b) The top 20 ranked VIP scores for NMR metabolite spectra peaks used by the PLS-DA model to distinguish infected from uninfected lungs. The length of the dashed line represents the relative value that each metabolite is given within the model.

**Fig. 4.**
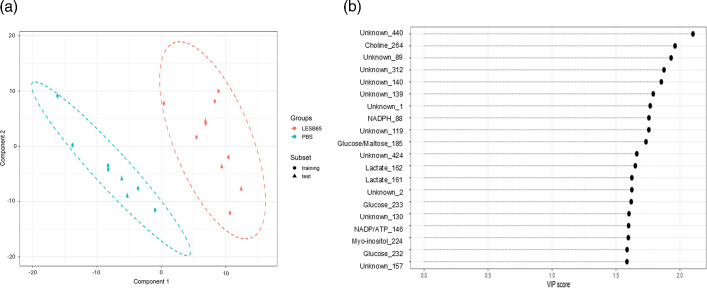
PLS-DA of sinus metabolome during *P. aeruginosa* infection. (a) PLS-DA was carried out to determine predictive models that distinguish metabolite profiles of sinus tissue infected with LESB65 (red) vs sham-infected with PBS (blue). In this scores plot, the data are represented by two PLS-DA components, with the model fit to a random subset of data and evaluated on the remaining data (circles for training and triangles for test). The test examples are inside the clusters of training examples, indicating a good model fit. (b) The top 20 ranked VIP scores for NMR metabolite spectra peaks used by the PLS-DA model to distinguish infected from uninfected sinuses. The length of the dashed line represents the relative value that each metabolite is given within the model.

The metabolic changes induced by infection of sinuses and lungs were relatively modest. Of 442 metabolite peaks identified from the NMR spectra of airway tissue, only 6 (1.4%) and 44 (10%) were significantly altered in abundance during infection in sinuses and lungs, respectively. Nonetheless, we reasoned that there may be more subtle underlying patterns in the data that would be suggestive of reproducible and predictable metabolic responses to infection. Using PLS-DA, a dimensionality reduction approach analogous to supervised PCA, we sought to develop predictive models that could distinguish infected from uninfected tissue. We applied the approach separately to sinus and lung NMR spectra, building two component models for both that had cross-validation area under the receiver operating characteristic curve (AUROC) scores of 1, indicating excellent model performance in distinguishing spectra derived from infected and uninfected tissue (File S2). Both models successfully predicted whether a subset of test tissue samples was derived from infected or uninfected mice, based on NMR spectra peaks (Figs3a  4a). VIP scores, describing the weighting given to each metabolite peak in the models, showed that the lung model included peaks associated with GSH, glucose and myo-inositol but also relied heavily on peaks for which metabolite identities are not yet described (Fig. 3b). Importantly, 8 of the top 10 weighted peaks, and 11 of the top 20, were associated with metabolite peaks with unknown identities. Similarly, the sinus model included 11 metabolite peaks of unknown identity amongst the top 20 (Fig. 4b). Notably, of the top 20 weighted peaks used by each model, only 3 were shared, reinforcing the notion that the metabolic shifts taking place in sinuses and lungs during infection are largely distinct.

## Discussion

Detailed profiling of airway samples from people with respiratory disease and experiencing chronic infection has yielded important information about the proteome, transcriptome and metabolome in disease [21]. One challenge in using sputum or other respiratory samples, however, is that it is often not possible to separate out material derived from the upper and lower airways. Mouse models enable us to do that, with post-mortem sampling of sinus and lung tissue enabling recovery of matched but discrete samples. The obvious caveats are that there are differences (including metabolic differences) between the mouse and human airways [22] and mice do not naturally develop symptomatic chronic respiratory bacterial infections.

Various experimental mouse models exist for the study of chronic airway infection, including those that achieve long-lasting and symptomatic infection via the surgical implantation of bacteria embedded within agarose beads [23]. We opted not to use models of this type, as we reasoned the inflammatory cascades initiated by surgery might significantly influence the metabolic profile of the airways. Instead, we used a natural inhalation model that had the advantages of inducing simultaneous sinus and lung infection and of establishing long-term infection, but with the caveat that infection is not associated with overt disease signs [6,24]. Furthermore, the utility of this infection model is highly dependent upon the bacterial strain chosen. We used the LES isolate B65, which is a representative of a transmissible clonal lineage causing respiratory infections in people with CF. It has some adaptations to airway environments that may have influenced the results obtained here. However, genomic studies have shown that *P. aeruginosa* CF isolates share the broader population structure of the species, which is defined by two major clades [25]. Nonetheless, our data reveal that even sub-clinical infection by *P. aeruginosa* results in demonstrable changes to the airway metabolome and that those changes differ between the upper and lower airways.

It should be noted that the contribution of secreted metabolites, embedded in mucus or airway surface liquid, to the metabolic environment of the respiratory tract during infection may have been underrepresented here. Whilst we did not observe macroscopic changes in mucus production or nasal secretions in infected mice, it is likely that mucus viscosity or composition will be altered by infection. The preparation of airway samples for NMR, with a PBS rinse used to remove blood from extracted tissue, will have depleted some airway liquid and mucus from the samples. Future studies could use nasal wash, brushings or bronchoalveolar lavage to specifically assess the metabolic landscape of the airway surface.

We observed more pronounced metabolic changes in the lungs than in the sinuses. These findings are in line with the proposed role of upper airway environments as protected niches for bacterial colonization that are less prone to robust inflammatory responses than the lung environment [4]. One limitation of our approach is the inability to distinguish between host- and pathogen-derived metabolites. However, we hypothesize that the increased abundance of GSH in the lungs of infected mice could result from *de novo* microbial production of this tripeptide. GSH is a potent antioxidant that can function as a reversible reducing agent, a role which may be critical in defence against host immune pressures. Indeed, *P. aeruginosa* mutants that are defective in GSH biosynthesis have been shown to have attenuated virulence in an acute respiratory infection model [26].

The decrease in myo-inositol abundance during infection was most pronounced in the lung environment but was also detected in sinuses. This carbocyclic sugar plays important roles in both host and pathogen biology. Myo-inositol regulates critical innate immune functions, including macrophage phagocytosis and superoxide production [27,28], and macrophages exposed to *P. aeruginosa*-conditioned media show evidence of increased demand for inositol [29]. On the microbial side, inositol phosphates are used for iron transport by *P. aeruginosa* [30], and access to free iron is a major constraint on bacterial growth in infection contexts [31]. Thus, the relative depletion of myo-inositol in infected airways might result from host and microbial competition for this key resource.

Our previous NMR metabolomics study used an acute bacterial respiratory infection model with *S. pneumoniae*, alongside an upper airway colonization model with the same species [11]. The key shared finding between that study and the one presented here is the relative depletion of glucose in the infected airways. We previously argued that this was likely driven by a requirement for increased host glycolysis to maintain essential innate immune function in the inflamed lung. Our findings here suggest that this demand for glucose is a general feature of bacterial airway infections, observable even under conditions where infection is not associated with overt signs of inflammation. Beyond this, there were relatively few similarities in the effects of *S. pneumoniae* or *P. aeruginosa* infection on the airway metabolome. We previously described a gradient of decreasing myo-inositol abundance between upper and lower airways of uninfected mice [11], but whilst this was minimally perturbed by *S. pneumoniae* infection, it was altered significantly by *P. aeruginosa* infection. Collectively, the two studies highlight the extent to which metabolic changes during infection are driven by the biology of the pathogen, with the character and magnitude of change dependent upon the infecting species. Future work may uncover specific metabolic vulnerabilities that could be exploited, either to aid clearance of infection or to bolster host defences. In this regard, it was notable how many of the NMR peaks that changed significantly in abundance during *P. aeruginosa* infection did not have defined metabolite identities. Assigning metabolites to those peaks and determining the extent to which their abundance changes in human infection should be priorities for future research. It is likely that some of the unidentified peaks will be microbe-derived, as our metabolite reference library contains greater coverage for mammalian metabolites than microbial ones. None of the exclusively microbial metabolites present in the reference library (e.g. pyoverdine, shikimates) were identified at detectable levels in the sample extracts used here. There are significant challenges in identifying unknown metabolites, both in metabolomics in general and NMR specifically, due to the lack of well-defined metabolome databases for non-mammalian organisms and the wide diversity in chemical structure and function of metabolites [32,35]. MS-based metabolomics could build on the findings we outline here. We opted for an NMR approach as a non-destructive method that is well suited to characterizing complex metabolite mixes, including whole tissue samples, and to profiling changes in such samples. It also affords advantages in terms of high reproducibility and low per-sample costs, with the benefit of allowing follow-up confirmatory experiments on the same sample to identify metabolites of interest. However, the sensitivity of MS approaches, combined with the ability to conduct targeted analysis, makes it a more suitable technology for follow-up studies that aim to explore the functional significance of the airway metabolic changes during infection that have been outlined here. It is anticipated that the data available in the public repository alongside this study will be of use to future research in this area, as these challenges are overcome by the concerted efforts of the microbial metabolomics community.

## Supplementary material

10.1099/mic.0.001617 Uncited Supplementary Material 1.

10.1099/mic.0.001617 Uncited Supplementary Material 2.

## Abbreviations

AUROC: area under the receiver operating curve
CF: cystic fibrosis
LB: lysogeny broth
LESB65: Liverpool Epidemic Strain B65
PCA: principal component analysis
PLS-DA: partial least squares discriminant analysis
PQN: probabilistic quotient normalization
VIP: variable importance in projection
