# Supplementary material for: The influence of Pseudomonas aeruginosa infection on the airway metabolome
Source: Microbiology (Reading). 2025 Oct 8;171(10):001617. doi: 10.1099/mic.0.001617 (PMC12507523; doi:10.1099/mic.0.001617)
Supplement: Uncited Supplementary Material 1. [file mic-171-01617-s001.pdf]

The influence of *Pseudomonas aeruginosa* infection on the airway metabolome

Green *et al*.

Supplementary Figures

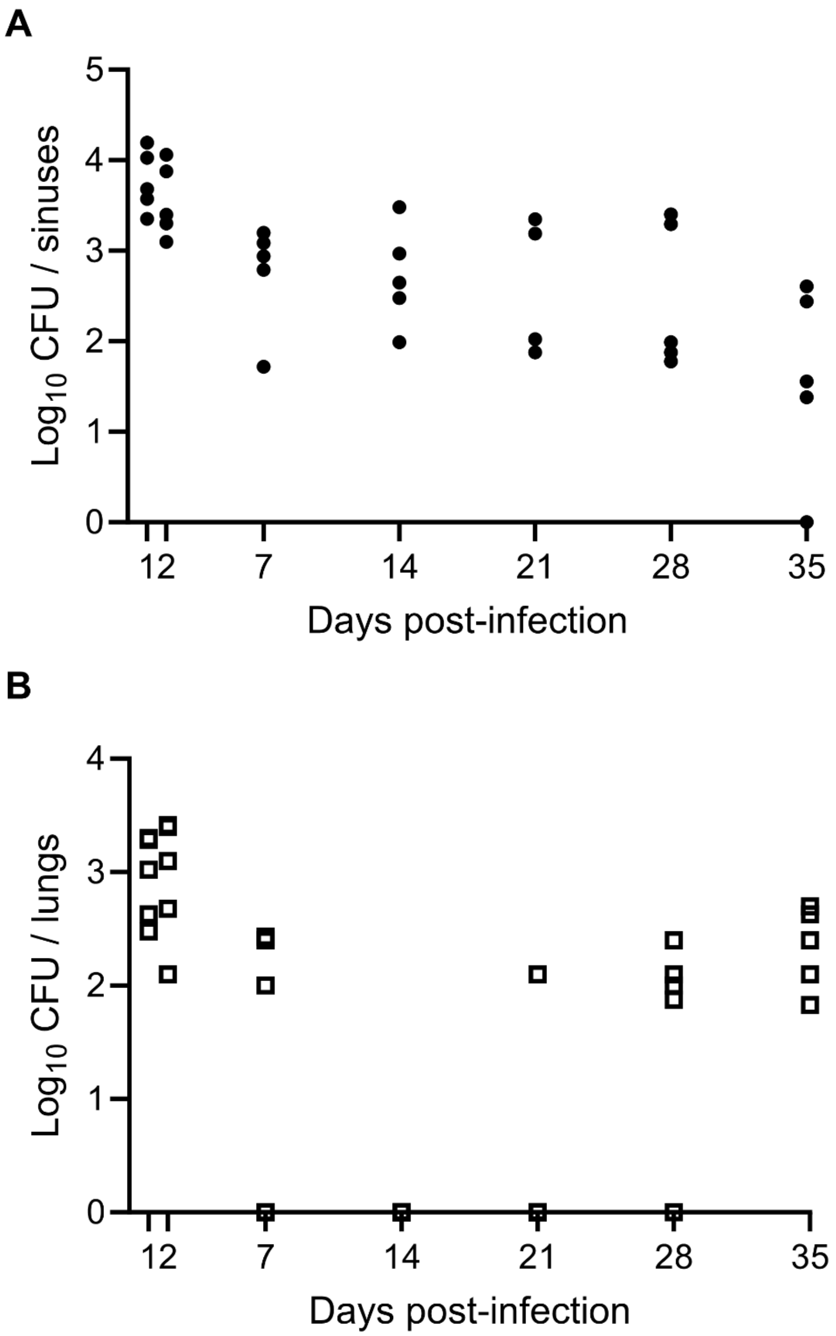

Supplementary Figure 1. Bacterial burden in airway tissues in a long term LESB65 infection model in mice. Colony forming units (CFU) in (**A.**) sinuses and (**B.**) lungs over 35 days of infection. BALB/c mice were infected with 2 x 106 CFU of LESB65 in saline, via intranasal administration. Each datapoint is derived from an individual mouse, with n=5 per timepoint.

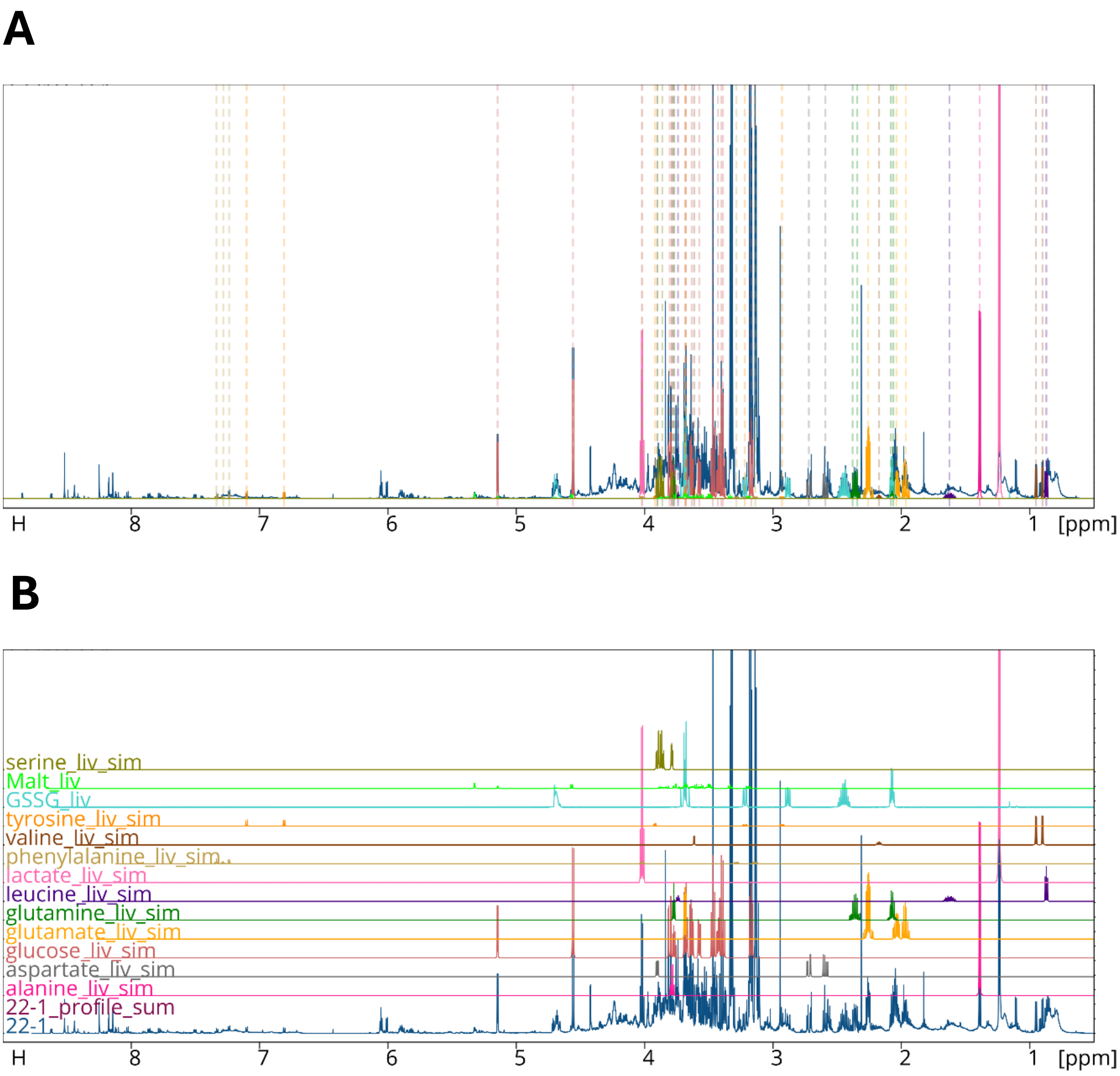

Supplementary Figure 2. An example NMR spectra with metabolite standards overlaid. (**A.**) Example infected lung extract spectra (dark blue) overlaid with several key metabolites (various colours). Dotted lines indicate the centre of each metabolite peak. (**B.**) Lung extract spectra (dark blue) stacked with several key metabolites (various colours). All spectra and standards were plotted using CcpNmr AnalysisMetabolomics (www.ccpn.ac.uk).

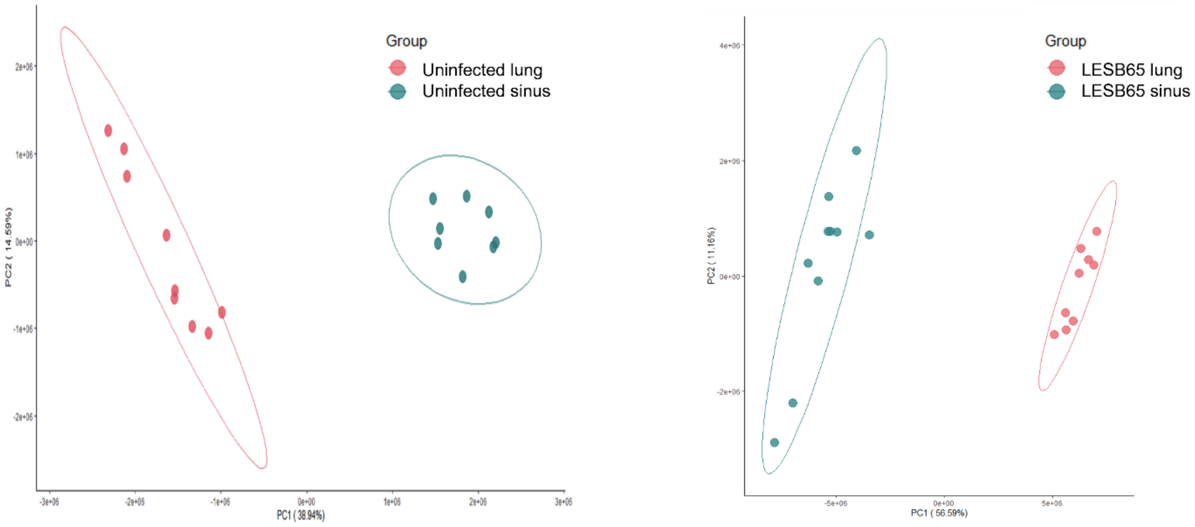

Supplementary Figure 3. Principal component analysis of sinus and lung tissue in uninfected (PBS intranasal) and LESB65-infected mice. All samples are at 48 hours post intranasal administration of PBS or bacteria. Each data point represents data from an individual mouse. Ellipses indicate a 95% confidence level. Data from uninfected animals were first reported in Green *et al*., PLoS Pathogens, 2023.

Supplementary Table 1. Principal component analysis of NMR spectra from infected and uninfected mouse airway tissue. Analysis conducted in R.

Dataset	Principal Component	% Variance Explained
Infected and uninfected sinus	1	52.54
	2	14.90
	3	11.98
	4	5.58
	5	4.85
	6	2.67
	7	2.13
	8	1.60
	9	1.10
	10	0.99
	11	0.63
	12	0.32
	13	0.30
	14	0.18
	15	0.12
	16	0.08
	17	0.04
Infected and uninfected lung	1	60.33
	2	15.56
	3	12.31
	4	5.63
	5	2.82
	6	0.82
	7	0.77
	8	0.48
	9	0.35
	10	0.32
	11	0.21
	12	0.13
	13	0.10
	14	0.06
	15	0.06
	16	0.03
